# Permeability Changes of Integrin-Containing Multivesicular Structures Triggered by Picornavirus Entry

**DOI:** 10.1371/journal.pone.0108948

**Published:** 2014-10-09

**Authors:** Pan Soonsawad, Lassi Paavolainen, Paula Upla, Wattana Weerachatyanukul, Nina Rintanen, Juan Espinoza, Gregory McNerney, Varpu Marjomäki, R. Holland Cheng

**Affiliations:** 1 Department of Molecular and Cellular Biology, University of California Davis, Davis, California, United States of America; 2 Department of Biological and Environmental Science/Nanoscience Center, University of Jyväskylä, Jyväskylä, Finland; 3 Department of Anatomy, Faculty of Dentistry, Mahidol University, Bangkok, Thailand; UMR Inserm U1052/CNRS 5286, France

## Abstract

Cellular uptake of clustered α2β1-integrin induces the formation of membrane compartments that subsequently mature into a multivesicular body (MVB). Enhanced internalization mediated by clustered integrins was observed upon infection by the picornavirus echovirus 1 (EVI). We elucidated the structural features of virus-induced MVBs (vMVBs) in comparison to antibody-induced control MVBs (mock infection) by means of high-pressure cryo fixation of cells followed by immuno electron tomography during early entry of the virus. Three-dimensional tomograms revealed a marked increase in the size and complexity of these vMVBs and the intraluminal vesicles (ILVs) at 2 and 3.5 hours post infection (p.i.), in contrast to the control MVBs without virus. Breakages in the membranes of vMVBs were detected from tomograms after 2 and especially after 3.5 h suggesting that these breakages could facilitate the genome release to the cytoplasm. The in situ neutral-red labeling of viral genome showed that virus uncoating starts as early as 30 min p.i., while an increase of permeability was detected in the vMVBs between 1 and 3 hours p.i., based on a confocal microscopy assay. Altogether, the data show marked morphological changes in size and permeability of the endosomes in the infectious entry pathway of this non-enveloped enterovirus and suggest that the formed breakages facilitate the transfer of the genome to the cytoplasm for replication.

## Introduction

Echovirus 1 (EV1) is a human pathogen and a member of the *Picornaviridae* family. EV1 binds to the α2β1-integrin transmembrane receptor on the plasma membrane of its host cell. This interaction is mediated through the I-domain of the α2 subunit and induces lateral redistribution and clustering of multiple receptors [1]. EV1 is rapidly internalized in complex with α2β1-integrin into caveolin-1 positive endosomes [2]–[4]. Recent data demonstrate that cellular entry of EV1 is not initiated from caveolin-1-enriched caveolar domains, but rather from the plasma membrane domains that are enriched in glycosylphosphatidylinositol (GPI)-anchored proteins, and that entry depends on regulators of macropinocytic uptake [5]–[7]. Tubulovesicular structures induced by clustering of α2β1-integrin at the plasma membrane eventually develop into α2-integrin triggered multivesicular bodies (MVBs) by growing intraluminal vesicles (ILVs) [5]–[7]. Viral capsid proteins and RNA remain within virus-induced MVBs (vMVBs) until initiation of replication, approximately 2.5 hours post-infection (p.i.) [3], [8]. Using live cell fluorescent staining of acidic endosomes [3], [5] as well as more accurate intra-endosomal pH measurement [6], we have demonstrated that these MVBs are not acidic compartments, unlike the endosomes of the clathrin pathway [9]. Other unique features of vMVBs include the lack of typical endosomal markers such as early endosome antigen 1, cation-independent mannose 6-phosphate receptor, CD63 and internalized transferrin, that altogether indicate that these vesicles are distinct from acidic late endosomes [2], [4], [5].

The mechanisms by which the genomes of non-enveloped viruses are translocated from the interior of the endosome to the site of replication are poorly understood. Results from studies of human rhinovirus 2, another picornavirus, suggest that the processes of uncoating and RNA penetration involve the low pH dependent generation of pores in the virus-containing endosome [10]. In contrast, poliovirus RNA is released from vesicles in the vicinity of the plasma membrane independent of endocytic acidification [11]. Similar to poliovirus, EV1, an acid-stable virus, may rely on mechanisms other than endosomal acidification for delivery of viral RNA into the cytoplasm. Our recent quantitative PCR results indicate that viral replication starts 3 hours p.i. [8]. However, uncoating of EV1 may appear as early as 30 minutes p.i., which has been shown by the appearance of the empty 80S-form virus particles in the infected cells after sucrose gradient sedimentation at different internalization time points [2]. In the current study, we characterized the changes of vMVBs based on structural and statistical analyses. Both two-dimensional (2D) and 3D transmission electron microscopic approaches were employed to explore the membrane integrity of MVBs and ILVs in detail. In addition, the cellular structures were preserved through a high-pressure freezing (HPF) and freeze substitution (FS). Compared to the mock infection, EV1 infection induced significant changes in size and membrane integrity of vMVBs, with a concomitant enlargement of ILVs. Specifically, ILVs began to exhibit breakages at 2 hours p.i., which was accompanied by rupturing of the limiting membrane of vMVBs. We propose that these breakages are associated with EV1 genome egress from the vMVBs and that they facilitate efficient replication.

## Materials and Methods

Cell Culture, Viral Infection and α2-integrin clustering. Experiments were performed using a human osteosarcoma cell line, overexpressing the α2-integrin subunit (SAOS-α2β1 cells) [12]. EV1 (Farouk strain; American Type Culture Collection) was produced and purified as described previously [2]. For visualizing the internalized virus and α2β1-integrin, all the following steps were conducted on ice unless otherwise stated in order to enable simultaneous internalization. SAOS-α2β1 cells were incubated with EV1 at 100 multiplicity of infection (MOI) for 1 hour. After several washes, the cells were incubated with EV1 antiserum for 1 hour and washed again. The cells were further treated with protein-A gold coupled with 6 and 14 nm gold particles (G. Posthuma and J. Slot, Utrecht) prepared according to [13] for 1 hour, followed by washing. The 6 nm gold particles were bound to α2β1-integrin and 14 nm gold particles to EV1. To eliminate nonspecific binding, the cells were treated with uncoupled protein-A (0.1 mg/ml) for 15 minutes and washed. Thereafter, the cells were incubated with monoclonal anti-α2-integrin antibody (Α211E10, from Dr. Fedor Berditchevski, Institute of Cancer Studies, Birmingham, UK). After washing, the cells were incubated with rabbit anti-mouse IgG (Sigma) for 1 hour, washed, and treated with protein-A gold (6 nm gold particles). The cells were then transferred to 37°C and incubated for 15 minutes, 2 and 3.5 hours. For visualizing the internalized α2β1-integrin only, (mock infection), the cells were incubated with monoclonal anti-α2-integrin antibody (Α211E10) and subsequently with rabbit anti-mouse IgG and protein-A gold (14 nm gold particles) under the same conditions as described above. These pre-labeled cells were further processed for HPF and FS as described below.

Confocal laser scanning microscopy. SAOS-α2β1 cells were incubated with monoclonal anti-α2-integrin antibody (A211E10) for 45 minutes on ice. After washing, the cells were further treated with Alexa Fluor 555 conjugated anti-mouse IgG (Invitrogen, Carlsbad, CA) for 45 minutes and washed again. In case normal integrin distribution was studied, without clustering, IgG Fab fragment DyLight 549 (Jackson ImmunoResearch Europe Ltd.) was used instead of anti-mouse IgG. When used, EV1 was added on cells along with the secondary antibody before labeling. Infection time began as the cells were removed from the ice and heated to 37°C by both a heater and hair dryer. The hair dryer was mounted in a stative close to the confocal stage and warm air was pointed towards the sample. The temperature inside the sample cuvette was tested with a miniature termometer to raise into correct temperatures. To compare α2β1-integrin levels in SAOS-α2β1 and A549 (expresses α2β1-integrin endogenously) cells, the cells were fixed with 4% paraformaldehyde for 20 min at room temperature, permeabilized with 0.2% Triton-X 100 for 5 min and stained for α2-integrin as described above. Confocal images were taken on an Olympus FluoView-1000 microscope (Olympus, CenterValley, PA) using a 543 nm excitation laser source, Alexa Fluor 555 filter settings, and a 60×1.35 NA oil objective. A sample size of 106×106 µm was scanned at 512×512 pixels using 2 µs/pixel scanning time with various laser transmissions and detector voltages. A total of 20–23 z-steps (0.5 µm each) was applied to image the entire sample area in 30 seconds Four different areas were imaged at once in 20-minute cycles for over 2.5 hours. Images were maximum intensity projected and mapped using ImageJ (NIH, Bethesda, MD).

High-pressure freezing (HPF) and freeze substitution (FS). Pre-labeling of SAOS-α2β1 cells for TEM, HPF and FS, as well as electron tomography, was performed according to our recently described methods [14]. The pre-labeled EV1- and mock- infected cells were loaded in flat specimen holders of an electron microscopy PACT HPF station (Leica Microsystems, Vienna) to be processed for high-pressure freezing, and transferred to liquid nitrogen. Both samples were freeze-substituted in 0.2% glutaraldehyde and 0.1% uranyl acetate in acetone at −90°C for 72 hours, and then warmed up slowly to −20°C (AFS; Leica Microsystems). After rinsing in acetone, the cells were infiltrated with gradually increasing concentrations of Lowicryl:ethanol (1∶3, 1∶1, 3∶1) and finally with pure resin. Each infiltration step took at least 12 h. The final polymerization was performed at −50°C using a UV light. Serial sections (80 nm–150 nm) were collected on Formvar coated, carbon-stabilized one-slot copper grids.

Electron microscopy and axial tilt tomography. TEM micrographs were collected using a JEOL 1230 operated at 120 kV. The electron dose for each image was 500–1000 e^-^/nm^2^, and the micrographs were digitized with a pixel resolution of 2048×2048 CCD camera. The measurements of MVBs were performed using the digitized images based on calibrated pixel size. After image screening, the data acquisition of tomography was done using a JEOL 2100F with the beam illumination of a field emission gun operated at 200 kV [14]. The recording and reconstruction pixel size was 1.0 nm and the electron dose per image was 500 e^-^/nm^2^. Projections were collected at one-degree tilt interval between −65° and +58° at the magnification of 10,000 using an automated software that utilized the cross-correlation algorithm to center the images with a pixel resolution of 4096×4096 CCD camera (TVIPS) (Video S1). Image alignment was done using cross-correlation algorithm based on 14 nm gold particles as fiduciary markers for this alignment. 3D reconstruction was done using weighted backprojection method, and validated with statistical reconstruction method (Fig. S1). After reconstruction, the volume rendering was done by Amira 4.1 framework (FEI) for density interpretation. The data is available at our supplementary data site: http://pioms.ucdavis.edu/pone/mvb.

Area measurements and statistical analysis. For micrograph assessment, the features of ILVs and MVBs were first identified in each 2D-TEM micrograph. The membrane architecture of both vesicles was carefully analyzed on these micrographs before categorizing them into “intact” and “broken”. The perimeter line on each vesicle was drawn around the membrane boundary (Fig. S2) using iTEM (Soft Imaging Solutions) or Image-J software (NIH). With the assumption that these vesicles are spherical in shape, the radius and thus areas of both MVBs and ILVs were calculated. The average areas between samples were compared statistically using a binomial t-test. A cut-off threshold value (α) between intact and broken ILVs was empirically quantified. All area measurement data from ILVs in each time point (>100) were pooled and re-analyzed statistically against this α-value. The vesicular areas above that α-value were found “broken”, while those below the α-value were “intact”. Percentage of successful prediction using this α-value relied on comparison between the percentage of “broken” ILVs retrieved from micrograph assessment (per total ILVs ×100) versus the percentage of those ILVs having their average areas more than the α-value.

Neutral red labeled EV1 photosensitivity assay. The neutral red (NR) EV1 was produced in GMK cells that were infected in the presence of 10 µg/ml of NR, according to the method described [2]. The virus was released at 20 hours p.i. by freeze-thawing the cells three times. EV1 was harvested by centrifugation and was used for experiments without further purification. SAOS-α2β1 cells were grown on glass coverslips and incubated with NR-EV1 for 1 hour on ice. The cells were then washed and incubated at 37°C. At the specific p.i. time points, the cells were exposed to white light for 10 minutes at room temperature and subsequently transferred to 37°C. The control cells were kept in darkness during the whole experiment. At 7 hours p.i., the cells were fixed with 4% paraformaldehyde for 20 minutes at room temperature. For immunofluorescence labeling, the cells were permeabilized with 0.2% Triton X-100. Of the cells stained with EV1 antiserum, the proportion of infected cells was calculated out of the total number of cells.

In vitro permeability test of α2-MVBs. EV1 was first bound on cells followed by incubation with antibodies against EV1 on ice. EV1 was then allowed to internalize for 1, 2 or 3 hours at 37°C and then placed on ice again. Surface EV1 was detected by goat anti-rabbit antibodies conjugated with Alexa Fluor 633. Then, the cells were permeabilized by placing the coverslip on a metal plate at -80°C for 20 seconds according to the protocol originally described by Robinson and Kreis [15]. Trypan blue was used to monitor the minimal time permeabilizing the plasma membrane. Cells were then immediately incubated with monovalent goat anti-rabbit IgG Fab fragment DyLight 488 (Jackson ImmunoResearch Laboratories) on ice for 30 minutes. Control cells were treated with Fab antibodies without permeabilization. Total EV1 signal was monitored after permeabilization of control cells with 0.2% Triton X-100 for 5 minutes. Samples were fixed with 3% paraformaldehyde for 15 min on ice and viewed under a confocal microscope. Permeability of MVBs was observed as the intensity of the Fab DyLight 488 antibody signal subtracted with the control surface Fab DyLight 488 labeling detected without permeabilization.

In order to monitor the permeability of the mock-infected samples, anti-α2-integrin antibody (Α211E10) was added to cells on ice, followed by incubating with clustering rabbit anti-mouse antibodies. The integrin clusters were allowed to internalize for 1, 2 and 3 hours at 37°C and placed on ice. As described above, goat anti-rabbit IgG Fab fragment DyLight 488 was bound on cells with or without permeabilization and fixed. As a control, also αV-integrin was clustered and internalized for 1 hour and tested for the permeability for the intracellular αV-integrin positive endosomes.

## Results

**Figure 1:**
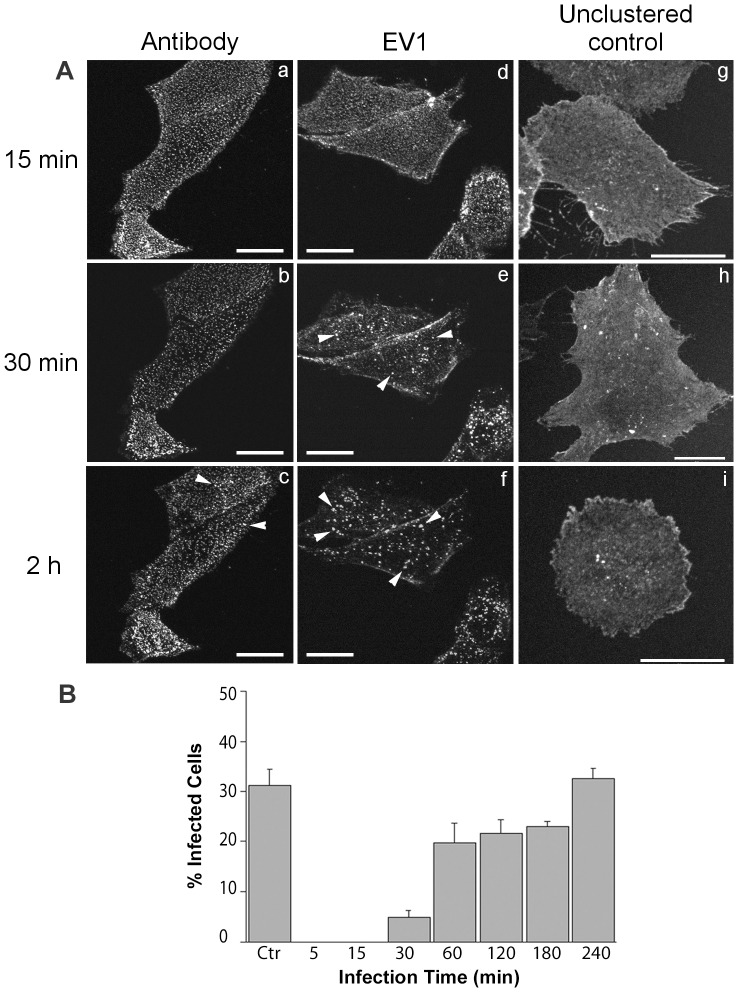
Time-course of α2β1-integrin internalization and viral uncoating upon EV1 infection. (A) Time-lapse confocal imaging of α2β1-integrin uptake for 15 minutes (upper panels), 30 minutes (middle panels) and 2 hours (lower panels) p.i. The uptake was stimulated by using antibodies (a, b, c) or EV1 (d, e, f) to cluster α2β1-integrin in SAOS-α2β1 cells. As a control for integrin clustering, α2β1-integrin distribution was followed for the same time periods after binding of non-clustering monovalent fluorescent Fab fragments (g, h, i), Scale bars: 20 µm. (B) Time-lapse uncoating status of EV1 evaluated by light-sensitive neutral red labeled virus. SAOS-α2β1 cells were treated with bright light for 10 minutes at indicated time points p.i. that rendered viral genome inside the capsid uninfectious and prevented further uncoating. At 7 hours p.i., the cells were fixed with 4% paraformaldehyde, stained with EV1 antiserum and the proportion of infected cells of the total cell number was calculated. Control cells were kept in darkness during the whole infection time interval. Example images are given for cells kept in the darkness for the whole period of 7 h (“DARK”) and for the cells given a 10 min light pulse (“LIGHT”) 10 min p.i. leading to a total block in infection.

EV1-induced integrin clustering leads to internalization and uncoating of EV1. SAOS-α2β1 cells were first labeled on ice to arrest the integrin movement and to synchronize clustering and internalization of α2-integrin, before warming up to 37°C on a microscope stage and imaged under a confocal microscope. Comparative analyses of α2β1-integrin clustering and internalization between EV1 and mock infections are shown in Figure 1. At ∼15 minutes p.i., punctate fluorescent signal was readily evident in the entire cytoplasm of both EV1 (Fig. 1A, (d)) and antibody-induced α2β1-integrin clustering (Fig. 1A, (a)). The result indicates that internalization of α2β1-integrin can be induced by both EV1 and integrin specific antibodies. At ∼30 minutes p.i., however, the larger fluorescent dots were found more notable in EV1-infected cells (Fig. 1A, (e), arrowheads) than those in the mock-infected cells (Fig. 1A, (b)). This increase of EV1-induced cluster size was found more pronounced at the infection time point of ∼2 hours p.i. (Fig. 1A, (f), arrowheads) and later (data not shown). In contrast, very few large fluorescent dots were visible at ∼30 minutes and 2 hours p.i. in the mock-infected cells (Fig. 1A, (b)).

As a control, the unclustered α2-integrin distribution was visualized by binding first the α2-integrin antibody and then the secondary Fab fragments conjugated with Alexa Fluor 555 (Fig. 1 A, (g,h,i)). Monitoring the labeled α2-integrin at 15, 30 and 120 minutes, α2-integrin was largely found on the plasma membrane and, to some extent, in scattered cytoplasmic vesicles during the 2 hours time period. Simultaneous internalization of transferrin-Alexa Fluor conjugate of these unclustered control cells showed colocalization of the internalized tubulo-vesicular structures with transferrin suggesting that normal α2-integrin internalization and recycling involves the transferrin recycling pathway, as similarly suggested for α2-integrins by others (see reviews [16], [17]).

We further evaluated the event of virus uncoating using light-sensitive neutral red labeled viral particles (Fig. 1B). Treatment of the cells with light for 10 minutes causes cross-linking of viral RNA, thus preventing any subsequent uncoating [11]. In contrast, uncoating of the RNA in the darkness and opening up the secondary structure of RNA releases the dye as was observed previously [11], and thus prevents further cross-linking, allowing infection to proceed. Therefore, the extent of infection after light treatment at different time points would represent the abundance of already uncoated virus particles. The results showed that capsid uncoating started at 30 minutes p.i. and it was greatly enhanced at 1 hour p.i. Infectivity after light treatment at 2–3 hours p.i. showed a comparable level of infection as that seen at 1 hour p.i. The results showed that integrin clustering by EV1 enhances cytoplasmic formation of large vMVB concomitant with early uncoating of the entered virus.

### Structural changes of ILVs and MVBs upon EV1 entry

**Figure 2:**
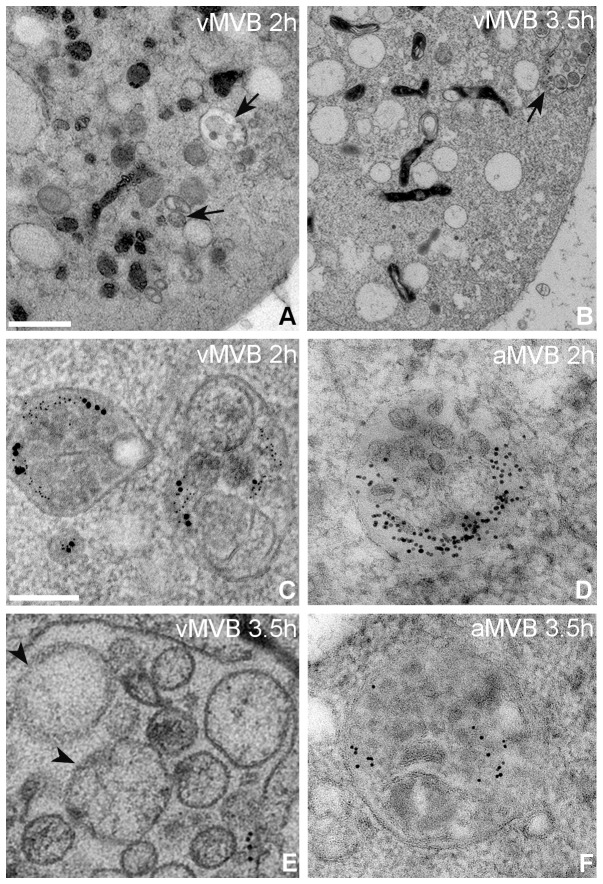
Morphological changes of MVBs and ILVs in the mock- and EV1-infected cells at the late infection periods. Clustering of α2β1-integrin in SAOS-α2β1 cells was induced on ice by EV1 that was followed by treatment with EV1 antiserum and protein-A gold (14 nm). After that, sequential binding of anti-α2-integrin antibody, followed by the clustering of secondary antibody and protein-A gold (6 nm), was performed and the cells were then incubated at 37°C for 2 (A) and 3.5 hours (B). Scale bars: 500 nm. For the mock infection, the cells were treated with anti-α2-integrin antibody followed by the clustering secondary antibody and protein-A gold (14 nm). The samples were further processed by HPF-FS, sectioned and viewed by TEM. The MVBs with internally located ILVs are marked by arrows (A, B). At the higher magnification, MVBs show detailed changes in their morphology and membrane irregularity at 2 hours p.i. (C) and particularly at 3.5 hours p.i. (E). The breakage of ILVs, evident by their disintegrated membranes (D, arrowheads) is notable at 2 hours p.i. Scale bars: 200 nm. The MVBs are small and round in shape in the mock-infected controls at both 2 and 3.5 hours p.i. (D and F).

Subcellular features of MVB morphogenesis was investigated with EV1 and α2-integrin being pre-labeled with corresponding antibodies and protein-A coupled to 14 nm and 6 nm colloidal-gold particles, respectively, in SAOS-α2β1 cells. With TEM at low magnification, a size increase of vMVBs was clearly visible at 2 hours p.i. (Fig. 2A, arrows). The enlargement of these vMVBs were even more pronounced at 3.5 hours p.i. (Fig. 2B, arrows), and appeared more irregular in shape at higher magnification. In contrast, antibody-induced MVBs were small and round-shaped at the same time point (Fig. 2, E and G). At 2 hours p.i., vMVBs showed large variation in the size and appearance of their ILVs. This allowed us to differentiate vMVBs into two populations – one with densely packed ILVs (Fig. 2C) and the other with loosely packed ILVs displaying size heterogeneity of the compartment (Fig. 2D). Interestingly, some large ILVs occasionally possessed fuzzy, blurred membrane boundary (Fig. 2D, arrowhead), suggesting membrane breakages. At 3.5 hours p.i., the majority of vMVBs were of the latter category, mainly filled with the large sized ILVs with heterogeneous electron density (Fig. 2F). In addition, large ILVs exhibited more blurred membrane features at this time point. In the mock-infected cells, there were no apparent structural changes or size increases in antibody-induced MVBs over a period of 3.5 hours p.i. Observation of mock-infected cells after 12 and 24 hours, however, revealed some breakages in their structures (data not shown). Apparently, antibody-induced clustering may exert similar changes, but with much slower pace compared to that of EV1-infected cells.

### ILV membrane breakage is associated with its size growth

**Figure 3:**
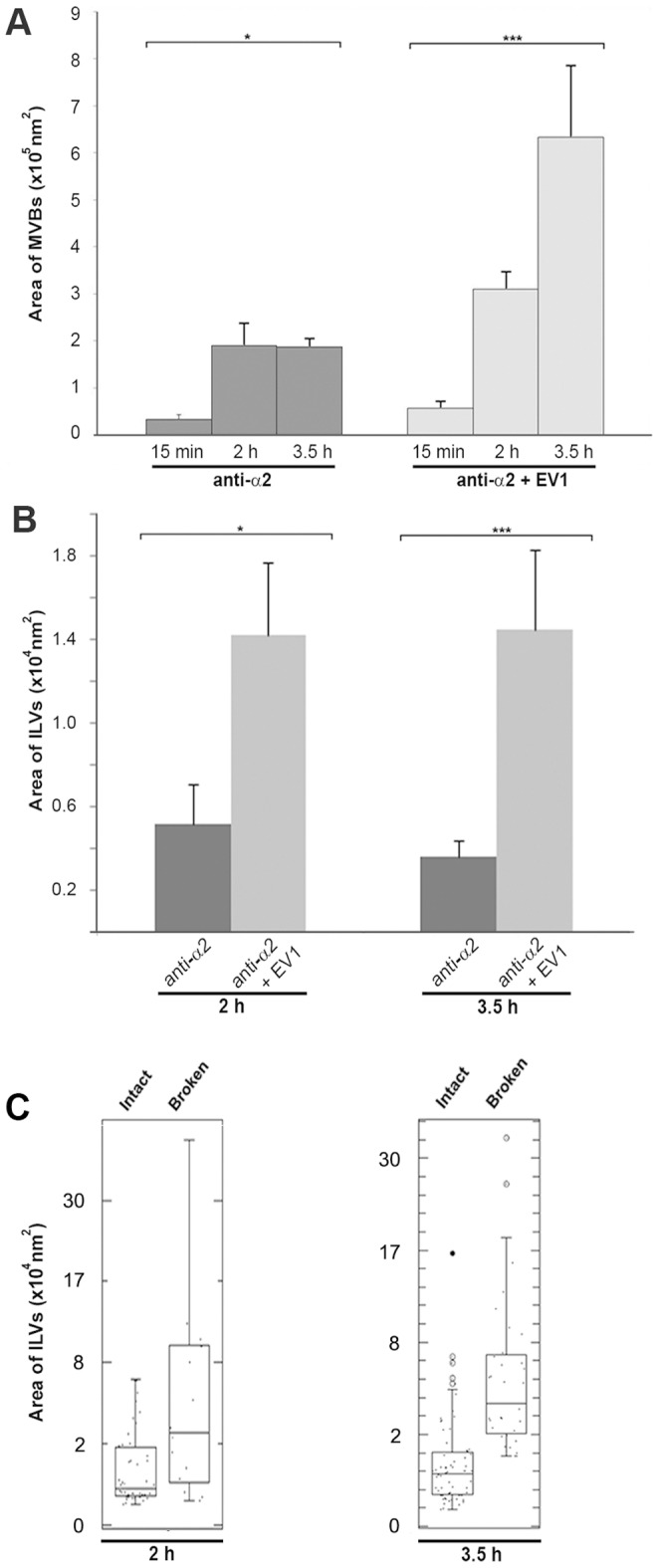
Significant growth of MVBs and ILVs induced by EV1. The area of MVBs and ILVs were drawn along their membrane perimeter in TEM micrographs and converted to areas. The deduced areas of MVBs and ILVs in both the mock (dark gray bars) and EV1 infection (light gray bars) were plotted against the time points post infection and expressed as mean±s.e.m (A and B). Asterisks denote significant differences (*, P<0.05 and ***, P<0.01) between the mock- and EV1-infected cells (A) and also between 2 and 3.5 hours p.i. in the EV1-infected cells (B). MVB average areas were calculated from 120 to 240 individual measurements and 200 to 300 individual measurements were used to calculate ILV average areas. (C) Analysis of the ILV areas in EV1-infected cells revealed two populations: intact and broken ILVs. The areas of intact and broken ILVs are expressed as mean±s.e.m.

To quantify the growth of vMVBs and ILVs during EV1 infection, the cross-sectional areas of these objects were calculated by drawing a boundary around the MVB and the containing ILVs in the EM micrographs, and converted into radii and areas. The error caused by variation between measured and maximum cross-sectional areas of objects was minimized with large number of measured objects, and was expected to be similar in different samples. Throughout the time study of EV1 infection, vMVBs progressively and significantly (P<0.01) expanded in the infected cells. The measured areas varied from ∼0.5×10^5^ nm^2^ (at 15 minutes p.i.) to 6.5×10^5^ nm^2^ (at 3.5 hours p.i.) (Fig. 3A). In the mock-infected cells, the areas of MVBs grew markedly (P<0.05) from 0.2×10^5^ nm^2^ to 1.9×10^5^ nm^2^ between the time points of 15 minutes p.i. and 2 hours p.i. However, there was no significant increase observed in the size of the vMVBs between 2 hours p.i. and 3.5 hours p.i. (Fig. 3A). Comparatively, the mean size of vMVBs in the EV1-infected cells was over three folds greater than that observed in the mock-infected cells at 3.5 hours p.i. In the case of ILV size, a marked difference was observed when the areas of ILVs were compared between the EV1- and mock-infected cells at 2 and 3.5 hours p.i. (Fig. 3B). The area increase of ILVs was found about three folds (1.40×10^4^ nm^2^ versus 0.50×10^4^ nm^2^) and four folds (1.45×10^4^ nm^2^ versus 0.38×10^4^ nm^2^) higher in the EV1-infected cells as compared to that of the mock-infected cells at 2 and 3.5 hours p.i., respectively.

We analyzed further the frequency of ILV membrane breakages during virus infection. At 2 hours p.i. and more prominently at 3.5 hours p.i., ILV membranes showed ruptured areas. The average areas of the broken ILVs were 4.98×10^4^ nm^2^ and 5.07×10^4^ nm^2^ at 2 and 3.5 hours p.i., respectively, while those of intact ILVs were ∼0.90×10^4^ nm^2^ at both time points (Fig. 3C). The results thus suggest that the large ILVs are more vulnerable to breakages possibly due to further enlargement in size.

### Disruption of ILV and MVB membranes as visualized by electron tomography

**Figure 4:**
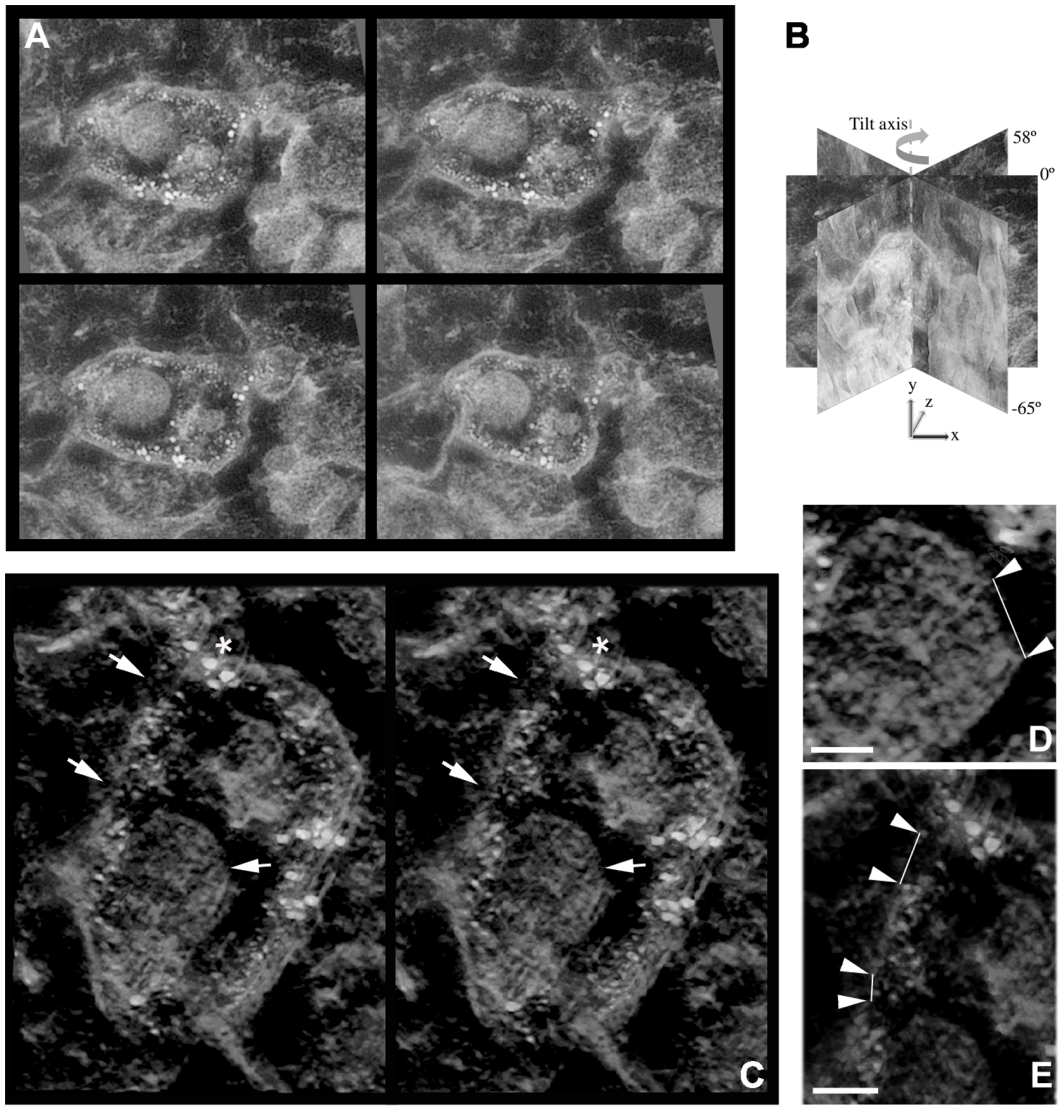
Membrane breakages of vMVBs and ILVs revealed by electron tomography. In the EV1-infected SAOS-α2β1 cells, areas of vMVB and ILV membranes were observed “broken” (C, D and E, white arrows) at 3.5 hours p.i. Projections (A, tilt angles from left to right, top to bottom: −33°, −11°, 11°, 33°) were collected at one-degree tilt interval between −65° and +58° (B) for 3D volume reconstruction and subsequent rendering shown in C. The stereogram of vMVBs were captured from the representative vMVBs with eight-degree seperation between the paired views. The inserts represent higher magnification of the rupture sites in the membrane of ILV (D) and vMVB (E) at 3.5 hour p.i. and correspond to the marked areas in C. Arrowheads indicate the distance of the rupture sites in the ILV and vMVB membrane structures measuring 76 nm (D) and 60 and 33 nm (E). Density of α2β1-integrin clusters were measured by manually drawing a region including vMVB membrane and a subregion including membrane near rupture sites. The measured density was 15.8% higher (22.0×10^−6^/nm^3^ and 19.0×10^−6^/nm^3^ gold particles near ruptures sites and in whole membrane, respectively) in the region including membrane near rupture sites. The measurements were consistent with reconstruction using a statistical method (Fig. S1). Scale bars: 50 nm (D and E).

To study the membranes of these vMVBs and their containing ILVs in further detail, three-dimensional image reconstruction of 120 nm-thick sections were revealed by electron tomography. At 2 hours p.i., the membranes of vMVBs remained largely intact. However, some ILVs, which showed blurred membrane morphology in projection images of the micrograph, clearly revealed membrane disintegration in the tomogram (data not shown). Intact ILVs were observed with the prominent edge and smooth membrane contour in the tomograms, representing complete membrane structures. At 3.5 hours p.i., the membrane architecture in vMVBs exhibited high irregularity with vesicular extensions. Although some of the large membrane ruptures could readily be distinguished on the projection images of the vMVB (Fig. 4A), other ruptured sites on the membrane were rather difficult to discern. In this regard, the volume rendering of the same vMVB revealed at least two distinct breakages on the limiting membrane as well as on the ILV membrane (Fig. 4, C, D and E, Fig. S1). The membrane breakages on both ILV and vMVB (at 3.5 hours p.i.) were clearly visible, with measurement of the membrane ruptures (Fig. 4, D-E, arrowheads), revealing opening sizes of 76 nm on ILV (Fig. 4D), and 33 nm and 60 nm on vMVBs (Fig. 4E). Moreover, α2β1-integrin and EV1, indicated by 6 nm and 14 nm gold particles, respectively, were co-localized underneath the vMVB limiting membrane and were measured to locate close to the rupture sites and ILV more likely than in other parts of the vMVB membrane. The average density of α2β1-integrin bound gold particles was 15.8% higher in volume including membrane rupture region than in volume including the whole membrane. The rupture sizes and density measurements were consistent with another reconstruction with a statistical method (Fig. S1). In the absence of EV1 as a control, antibody-induced MVBs were not associated with any breakages at all three time points and hence, these negative results have not been added in the figures here. However, much later at 24 hours p.i., despite the small size of the structures, some breakages were occasionally observed in their membranes (data not shown). The result suggests that membrane disintegration of antibody-induced α2β1-integrin internalization could only take place at much slower rate without the presence of EV1.

**Figure 5:**
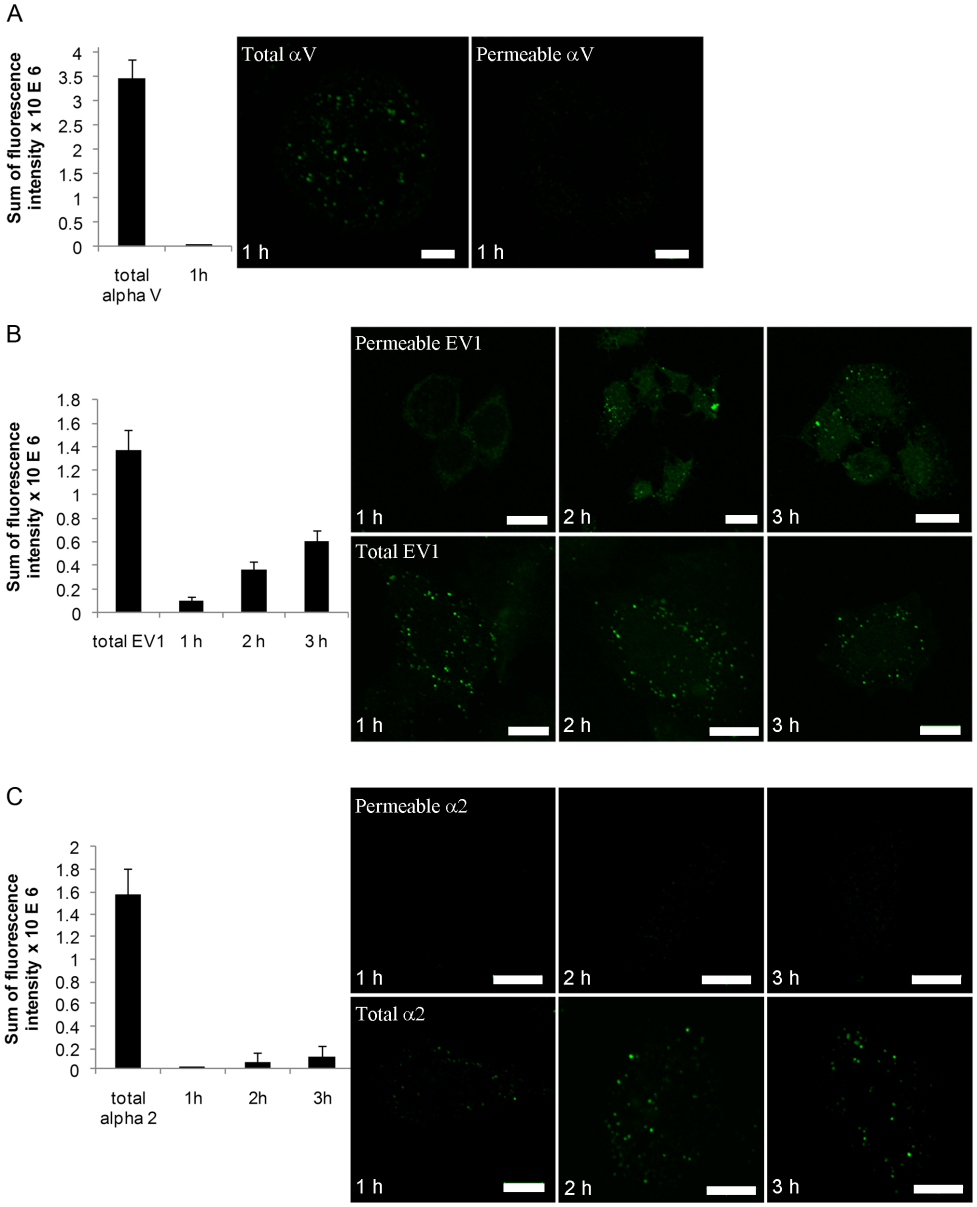
Permeability of MVBs increases during EV1 infection. (A) Control permeability measurement using αV integrin positive endosomes. Mouse antibodies against αV was bound on the cell surface followed by clustering with rabbit anti-mouse antibodies on ice. After 1 h internalization at 37C and subsequent permeabilization monovalent goat anti rabbit Fab fragments conjugated to DyLight 488 were allowed to bind for 30 min in ice and fixed. Fluorescence intensity of Fab fragments bound due to permeability of the endosomes was measured from confocal z-stacks. Surface label, measured from non-permeabilized control cells, was subtracted from the results of ice permeabilized cells. Total intensity refers to the fluorescence detected after permeabilization of all membranes with Triton X-100. Permeability was then measrued from samples treated with EV1 (B) or α2-integrin (C). Intensity of the goat anti rabbit Fab fragments conjugated to DyLight 488 was similarly measured from these samples. Example images showing permeability or total fluorescence are shown. Altogether 30 cells from three independent tests were analyzed. Results are mean values ±s.e.m. Bars, 10 µm.

Further, we developed an in vitro permeability assay to detect the presence of breakages in vMVBs observed by EM (Fig. 5). In this assay, the plasma membrane was gently permeabilized by rapid freezing, without affecting the cytoplasmic endosomal structures as described in Robinson and Kreis [15]. Trypan blue labeling also confirmed that the freezing protocol permeabilized the plasma membrane efficiently (data not shown). Permeability was monitored by the presence of a Fab anti-rabbit DyLight signal that was able to enter permeabilized endosomes and bind to intra-endosomal anti-EV1 antibodies. The anti-EV1 antibodies were originally bound on the plasma membrane before internalization and were thus in the lumen of the endosomes (Fig. 5). This method was controled by testing the permeability of other endosomal structures that were induced by αV-integrin clustering and internalization for 1 hour. Alpha V positive endosomes showed no apparent increase of permeability as expected (Fig. 5A). After EV1 induced internalization, permeability was low at 1 hour p.i., but increased clearly at 2 hours p.i. and became more obvious at 3 hours p.i. (Fig. 5B). We also evaluated the total amount of signal (anti-EV1) that was accessible to the Fab fragments by permeabilizing all cellular membranes with 0.2% Triton X-100. We observed that, at 3 hours p.i., approximately 40% of the total EV1 signal was reached by permeabilization caused by virus infection. In contrast, Fab DyLight signal of mock infection showed that the endosomes were not sufficiently permeabilized as the fluorescence intensity was rather low between 1 hour and 3 hours post internalization (Fig. 5C), with insignificant increase of permeability observed at 2 and 3 hours p.i.

## Discussion

**Figure 6:**
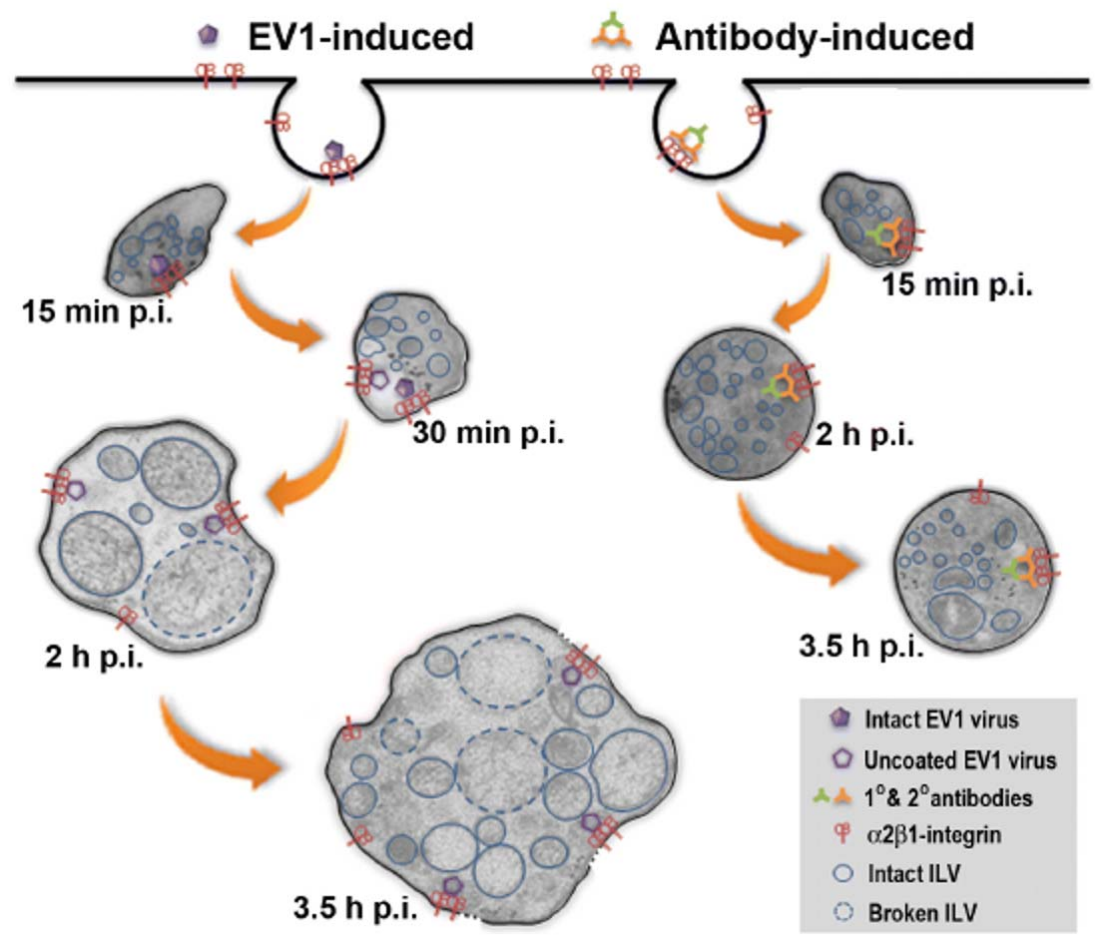
Pictorial illustration summarizing events during EV1 and mock infections. 1) Binding of EV1 to α2β1-integrins on the plasma membrane leads to clustering of integrins and internalization of virus-receptor complex through macropinocytosis. Within 15 min p.i., the internalized vacuoles start to enlarge in size and form internal vesicles giving rise to α2-MVBs. Inside the α2-MVBs, within the growing vesicles, EV1 uncoating of virus particles begins 30 minutes p.i., exposing the viral genomes to the intraluminal compartment of the vesicles. MVBs grow significantly in size 2 and 3 hours p.i. Within these α2-MVBs, ILVs show varying electron densities and grow until their membranes start to disintegrate at 2 hours p.i. This is concomitant with breakages of MVB limiting membranes that is more obvious at 3.5 hours p.i. Likely, this structural disintegration of MVBs provides EV1 the means to release the viral genome to the cytoplasm for replication. 2) In mock infection, binding of specific antibodies to α2β1-integrins causes clustering and internalization of integrins into the cytoplasm, as well. However, compared to the EV1 infection, the MVBs grow moderately, remain stable in shape and do not show enhanced disintegrating structural changes by 3.5 hours p.i.

The entry of EV1 to the host cells is triggered by the clustering of the α2β1-integrin molecules on the plasma membrane [18]. As a result, a novel endocytic structure, integrin specific MVB, is formed and serves as a portal for EV1 entry and facilitates subsequent replication (Fig. 6) [1], [3], [5], [8]. We show that EV1-induced structural changes of MVBs with detectable ruptures of the limiting membrane occur between 1 and 3 p.i., before the measured startup of replication in the cytoplasm [8]. However, the uncoating was observed to start already earlier suggesting that RNA may wait inside endosomes for the release to the cytoplasm. How is the viral RNA kept stable inside the vMVBs remains to be studied. TEM image analysis clearly showed two pools of MVBs in EV1-infected cells based on their morphology, namely loosely and densely packed MVBs. Loosely packed MVBs were not present in the mock-infected cells, indicating that EV1 contributes to the formation of such low-density feature in its resident compartmental structures. In contrast, the densely packed MVBs were mostly found in the early entry of EV1 or generally in the mock-infected cells, and they did not exhibit any apparent membrane breakages. The ruptures of ILV and MVB membranes were evident by ET starting at 2 hours p.i., and became more obvious at 3.5 hours p.i. in the EV1-infected cells, coinciding with the permeability change of vMVBs observed by fluorescence microscopy. Only 40% of the total EV1 signal that was taken up into endosomes was accessible to the cytoplasm-derived Fab fragments in the confocal microscopy assay. Likely, the development of permeable membrane in vMVB is one of the limiting factors in the viral infection process.

Endosomes are involved in protein sorting for degradation in lysosomes or for recycling back to the plasma membrane having implications in cell signaling and migration. Endosomes also serve as convenient means for virus entry or exit from the host cells. Numerous enveloped RNA viruses, including Lassa virus, utilize endosomes for budding [19]. Other viruses, such as VSV, deliver a nucleocapsid into the lumen of ILVs through fusion of the viral envelope with endosomal membranes [20]. Our structural observations suggest that α2β1-integrin and EV1 are not particularly sorted to the ILVs inside a vMVB. However, the importance of ILVs in EV1 infection is supported by our previous finding that the amiloride analog, EIPA, blocks EV1 infection [5]. EIPA caused accumulation of intracellular vesicles prematurely by inhibiting the ILV formation, and thus preventing the maturation of vMVBs. In addition, expression of the dominant negative Vps4, one key component of the ILV forming machinery, lead to the inhibition of EV1 infection [6] as well. In that same study, the intra-endosomal pH was assessed relatively neutral during the first 3 hours of internalization [6], the period before these vMVBs released the viral genome for replication [3]. These unique biochemical characteristics of vMVBs may dictate a novel mechanism of genome release compared to those via the clathrin-dependent pathway.

The α2β1-integrin clustering may directly or indirectly associate with the mechanisms leading to the ruptures of limiting membrane. To date, there have been limited reports available regarding the structural changes of endosomes induced by viruses. Our immuno-EM results indicated that, upon internalization induced by EV1, α2β1-integrins remain localized underneath the inner leaflet of vMVB membrane without recycling back to plasma membrane for at least 3.5 hours p.i. (Fig. 2). This is in line with our recent observation that the internalization of clustered α2β1-integrin molecules does not lead to the recycling of these molecules from the limiting membrane [21]. Instead, we observed that α2β1-integrins clustered close to the ruptured sites (Fig 4). Earlier, we found that neutral calcium-activated proteases, calpains, colocalized with vMVBs and that the presence of EV1 enhances calpain activation [8], [21]. Also, α2β1-integrin was found to undergo proteolytic cleavage by calpains [21], [22]. Moreover, these neutral calcium-dependent cysteine proteases have been shown to play a role not only in EV1 infections, but also in another enterovirus coxsackievirus B3 [8], [23]–[26]. Whether calpain proteases contribute directly to the above processes, which lead to the change of endosomal permeability and thus the rupture of limiting membrane, remains to be shown.

In conclusion, both EV1 and antibody cluster α2β1-integrin molecules, and lead to the biogenesis of multivesicular structures. Uncoating of EV1 starts at 30 minutes after entry, and approaches the plateau level at 1 hour p.i. The presence of EV1 leads to the size increase of these MVBs and the permeability increase of the MVB limiting membrane and of the ILVs inside the compartment. These ruptures may facilitate the release of the viral genome to the cytoplasm of host cell. The results provide us new insights of how a non-enveloped RNA virus may break down the endosomal barrier to facilitate replication in the cytoplasm.

## Supporting Information

Figure S1 **Comparing vMVB reconstruction with weighted backprojection to statistical reconstruction method.** Weighted backprojection (A, C, E) was used in the study for tomography reconstruction. It is well-known that backprojection methods are sensitive to missing wedge causing artifacts in the z-direction (Fig. 4 B, sample depth) of the reconstruction. To validate our findings, we applied recently developed statistical reconstruction method (B, D, F), sequential maximum a posteriori expectation maximization (sMAP-EM, unpublished method), to 3.5 hours p.i. vMVB projection data. Volume renderings of the reconstructions with BioImageXD software show that vMVB membrane breakages are clearly visible with both weighted backprojection (A, white arrows, zoomed region in C) and sMAP-EM (B, white arrows, zoomed region in D) reconstruction methods. The breakage in the ILV membrane is more easily visible in the sMAP-EM reconstruction (F, white arrows) but still detectable also in the weighted backprojection reconstruction (E, white arrows). Also, density measurements of α2β1-integrins bound to 6 nm gold particles were consistent. We can conclude that even though statistical reconstruction methods generally reduce artifacts in limited angle tomography as compared to backprojection methods, large vMVB and ILV membrane breakages and dense gold particles studied in this work were visible regardless of the reconstruction method.
(TIF)

Figure S2 **Manual measurements of ILVs.** ILVs were measured by drawing a boundary with interpolated polygon tool in iTEM software. The area and perimeter of closed contour is automatically calculated. In this example the area and perimeter of measured ILVs were 1180.57 nm^2^/130.15 nm (top), 1460.85 nm^2^/144.03 nm (left), and 1483.39 nm^2^/145.41 nm (right).
(TIF)

Video S1 **Aligned set of projections of vMVB for tomography study.** 124 projections were imaged at one-degree tilt interval between −65° and +58°.
(AVI)
