# Supplementary figures and images for: Permeability Changes of Integrin-Containing Multivesicular Structures Triggered by Picornavirus Entry
Source: PLoS One. 2014 Oct 9;9(10):e108948. doi: 10.1371/journal.pone.0108948 (PMC4191987; doi:10.1371/journal.pone.0108948)

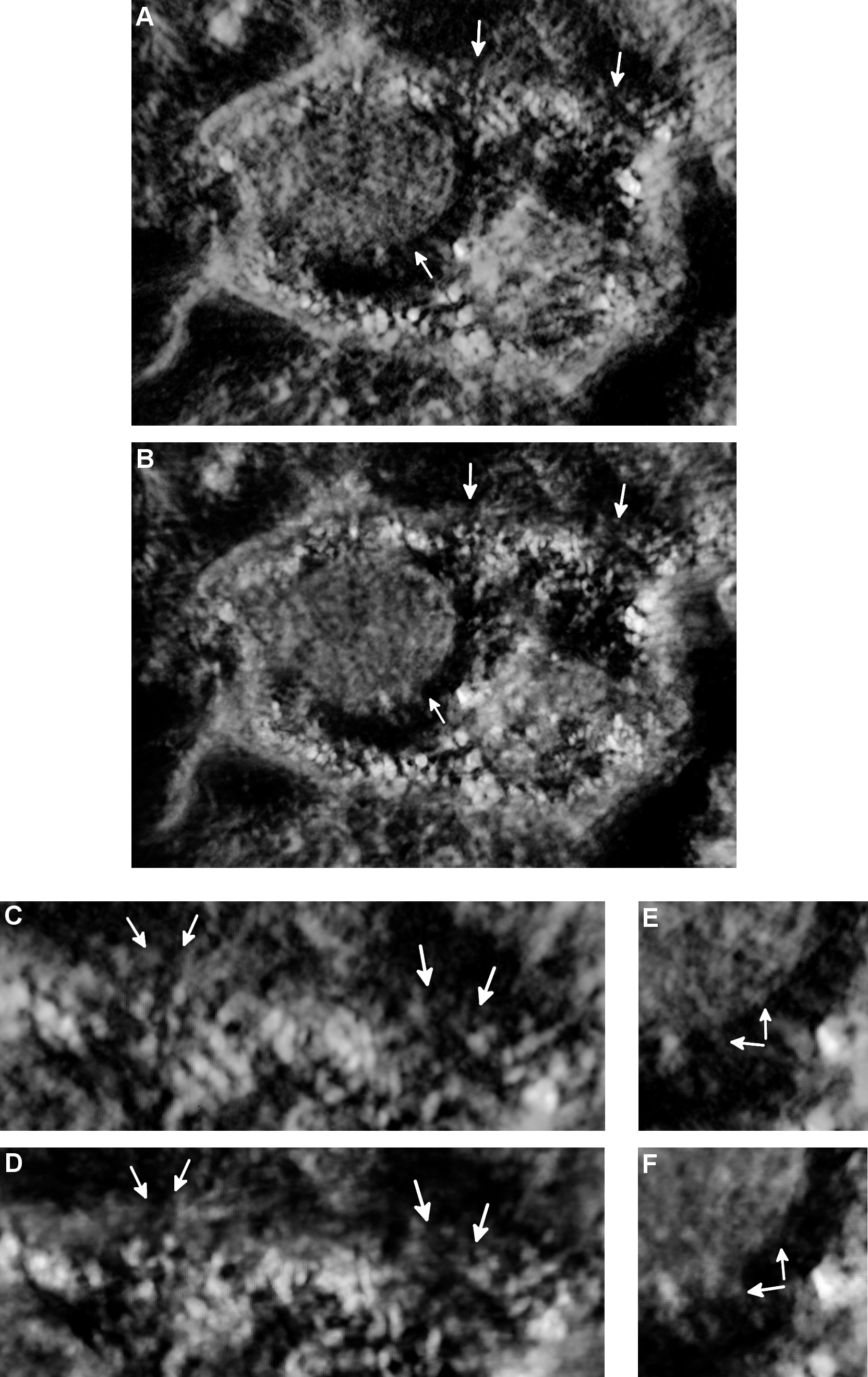

Supplement: Figure S1 — Comparing vMVB reconstruction with weighted backprojection to statistical reconstruction method. Weighted backprojection (A, C, E) was used in the study for tomography reconstruction. It is well-known that backprojection methods are sensitive to missing wedge causing artifacts in the z-direction (Fig. 4 B, sample depth) of the reconstruction. To validate our findings, we applied recently developed statistical reconstruction method (B, D, F), sequential maximum a posteriori expectation maximization (sMAP-EM, unpublished method), to 3.5 hours p.i. vMVB projection data. Volume renderings of the reconstructions with BioImageXD software show that vMVB membrane breakages are clearly visible with both weighted backprojection (A, white arrows, zoomed region in C) and sMAP-EM (B, white arrows, zoomed region in D) reconstruction methods. The breakage in the ILV membrane is more easily visible in the sMAP-EM reconstruction (F, white arrows) but still detectable also in the weighted backprojection reconstruction (E, white arrows). Also, density measurements of α2β1-integrins bound to 6 nm gold particles were consistent. We can conclude that even though statistical reconstruction methods generally reduce artifacts in limited angle tomography as compared to backprojection methods, large vMVB and ILV membrane breakages and dense gold particles studied in this work were visible regardless of the reconstruction method. (TIF) [file pone.0108948.s001.tif]

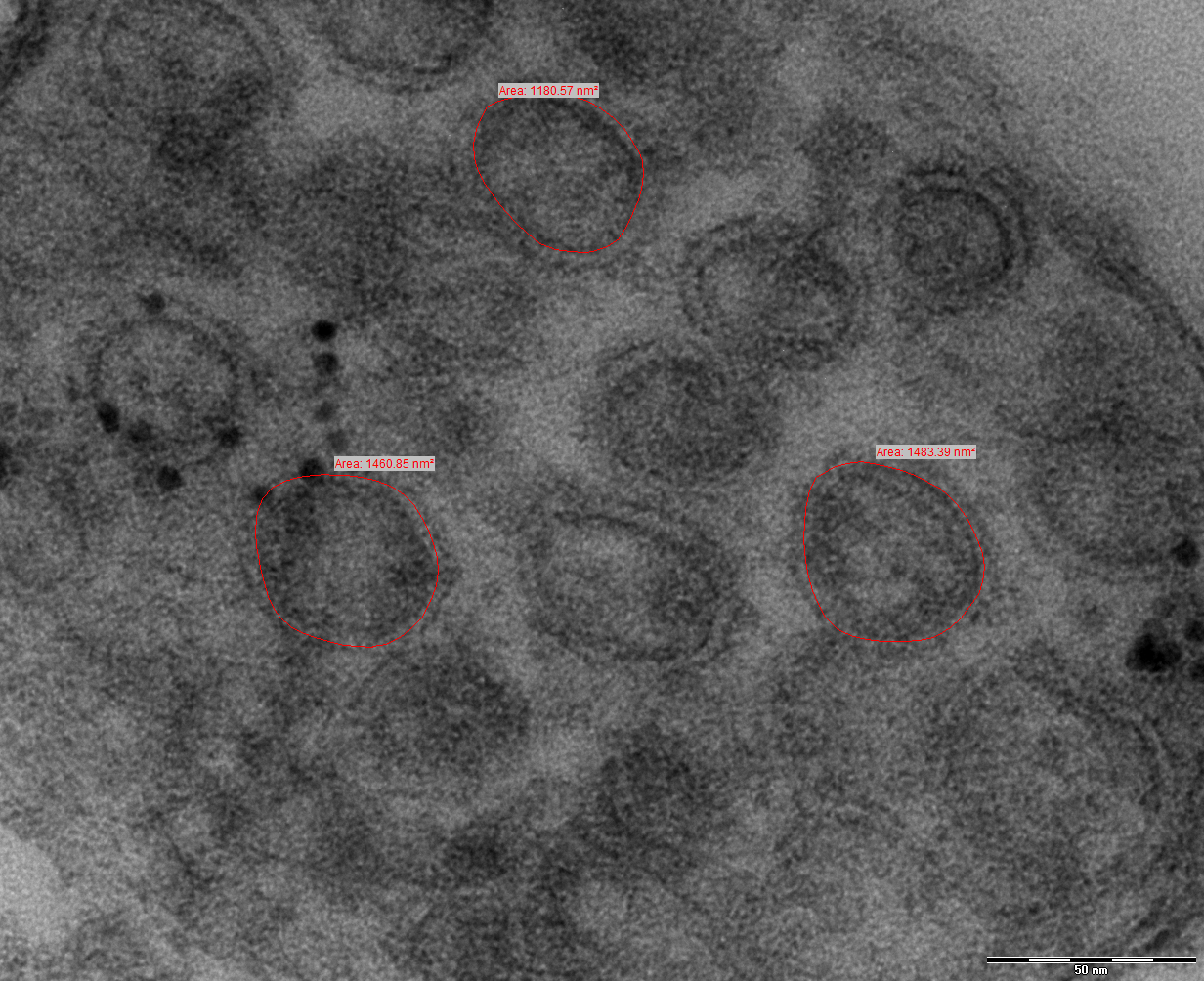

Supplement: Figure S2 — Manual measurements of ILVs. ILVs were measured by drawing a boundary with interpolated polygon tool in iTEM software. The area and perimeter of closed contour is automatically calculated. In this example the area and perimeter of measured ILVs were 1180.57 nm2/130.15 nm (top), 1460.85 nm2/144.03 nm (left), and 1483.39 nm2/145.41 nm (right). (TIF) [file pone.0108948.s002.tif]
